# Computer aided planning of patches and conduits for surgery in congenital heart disease

**DOI:** 10.1186/1532-429X-13-S1-P191

**Published:** 2011-02-02

**Authors:** Eugenié Riesenkampff, Michael Huebler, Urte Rietdorf, Tobias Heimann, Tobias Schwarz, Hans-Peter Meinzer, Felix Berger, Titus Kuehne

**Affiliations:** 1 German Heart Institute Berlin, Berlin, Germany; 2 German Cancer Research Center Heidelberg,Department of Medical and Biological Informatics, Heidelberg, Germany

## Introduction and purpose

To optimize outcome in congenital heart disease, the aim is to establish computer assisted methods for planning and simulating surgery in an objective and quantitative manner.

## Methods

Based on 3D MRI datasets, two applications were developed: (1) for surgical correction of hypoplastic aortic arches the calculation of vessel diameters, and a patch for optimal surgical correction. This application was evaluated on 12 test datasets and phantoms. (2) For assessing the feasibility of biventricular repair in the case of complex cardiac malformations, an application for simulation of intracardiac repair with a Rastelli like procedure was developed and tested.

## Results

**Figure 1:**
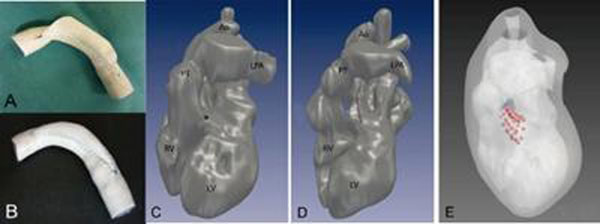


(1) In all test datasets with varying aortic arch pathologies, diameters were determined with the new application with minor differences (1.5 ± 1.2 mm) compared to the standard measurements. Individual patches were calculated. In phantoms (Goretex) of pathologic aortas, the patches were inserted successfully (Figure 1: Goretex phantom with partial (A) and fully (B) inserted patch). MRI thereafter revealed well-formed aortas. (2) Furthermore, an intracardiac conduit was computed for biventricular repair (Figure 1, panel C, D and E).

## Conclusions

The calculation of patch material for surgical reconstruction of aortic pathologies is possible from 3D MRI data and first preclinical tests were successful. For complex cardiac malformations, the preoperative evaluation of the operation method will be feasible. Further preclinical testing is needed.

